# Unveiling Neglected Pin-Site Tuberculosis: An Uncommon Encounter Due to Surgical Distal-End Radius Fracture Management With K-Wires

**DOI:** 10.7759/cureus.53986

**Published:** 2024-02-10

**Authors:** Hardik Patel, Dr Aditya Pundkar, Sandeep Shrivastava, Suyash Y Ambatkar, Saksham Goyal

**Affiliations:** 1 Department of Orthopaedics, Jawaharlal Nehru Medical College, Datta Meghe Institute of Higher Education and Research, Wardha, IND

**Keywords:** tuberculosis, infection, pin site, osteoarticular tuberculosis, extrapulmonary tuberculosis

## Abstract

In this case report, a 29-year-old man underwent surgery to treat a fracture to the left distal end of his radius using closed reduction and K-wire fixation. The patient was advised to follow up in the outpatient department after six weeks for cast and K-wire removal. Still, the patient failed to do so and was doing alternate day dressing of the K-wires. After six months he slipped and fell from his cot while sleeping, sustaining an injury to the left wrist. Initially, he developed a swelling over the wrist, which suddenly increased in size and ruptured. Thick white caseous material was leaking out from the wounds. The patient underwent debridement and K-wire removal. An intraoperative sample was sent for a bacterial culture sensitivity test, histological analysis, and cartridge-based nucleic acid amplification test (CB-NAAT/GeneXpert). Postoperatively, anti-tuberculous treatment was started. The patient fully recovered from tuberculosis and had a complete range of movements after treatment.

## Introduction

Among the serious health issues that developing nations have to deal with, tuberculosis stands out. Globally, around 27.1% of patients suffering from tuberculosis are from India. About 10% of instances of extrapulmonary tuberculosis are of the musculoskeletal variety, and 10% of patients with musculoskeletal disorders have hand involvement. The occurrence rate of wrist tuberculosis is infrequent. Skeletal involvement occurs in 1-3% of TB patients [1]. It has been noted that 51% of skeletal TB originates in the spine and extraspinal articulating joints [2]. Wrist tuberculosis following an infection of the pin site is a very rare presentation. Tuberculosis of the joints has a prolonged onset and is rarely diagnosed before developing into the stage of severe arthritis [1].

Poncet’s disease, or tuberculosis rheumatism, is distinguished from tuberculosis arthritis, as the former condition observed in the acute phase of tuberculosis consists of non-destructive variants of joint inflammation. On the other hand, tuberculosis arthritis manifests in a single joint from which the organism can be recovered [2]. Tuberculosis arthritis originates with symptoms of synovitis and leads to periarticular demineralization, marginal erosions, and eventually damage of the affected joint [3]. In overweight patients, faster progression can be seen, from synovial inflammation to damage of joints. In the manifestation of superinfection (e.g., *Staphylococcus aureus*), the hastening of joint destruction occurs with the involvement of a systemic inflammatory response [4].

Delayed diagnosis of tuberculosis is largely attributable to the non-directional, misleading presentation of patients with malaise, anorexia, and other constitutional symptoms [5]. Delays in the diagnosis and treatment of the mycobacterial infection can lead to the destruction of larger portions of the bones, adjacent bones, or joints [6]. Thus, the manifestation, diagnosis, and management of treatment should be understood thoroughly.

## Case presentation

A 28-year-old male patient presented to the orthopedics outpatient department of Aacharya Vinoba Bhave Rural Hospital, Sawangi, with pain and discharge from a wound over his left wrist. The patient reported an initially uncomplicated injury to his left wrist caused by falling from his wooden cot while sleeping at his home three days prior. Immediately, after the fall, he experienced pain, which was initially dull and aching gradually becoming progressive. It was associated with swelling, which grew over time and ruptured. A whitish discharge was seen oozing out of the wound. There was no history of fever, loss of weight, loss of appetite, or evening rise in temperature.

**Figure 1:**
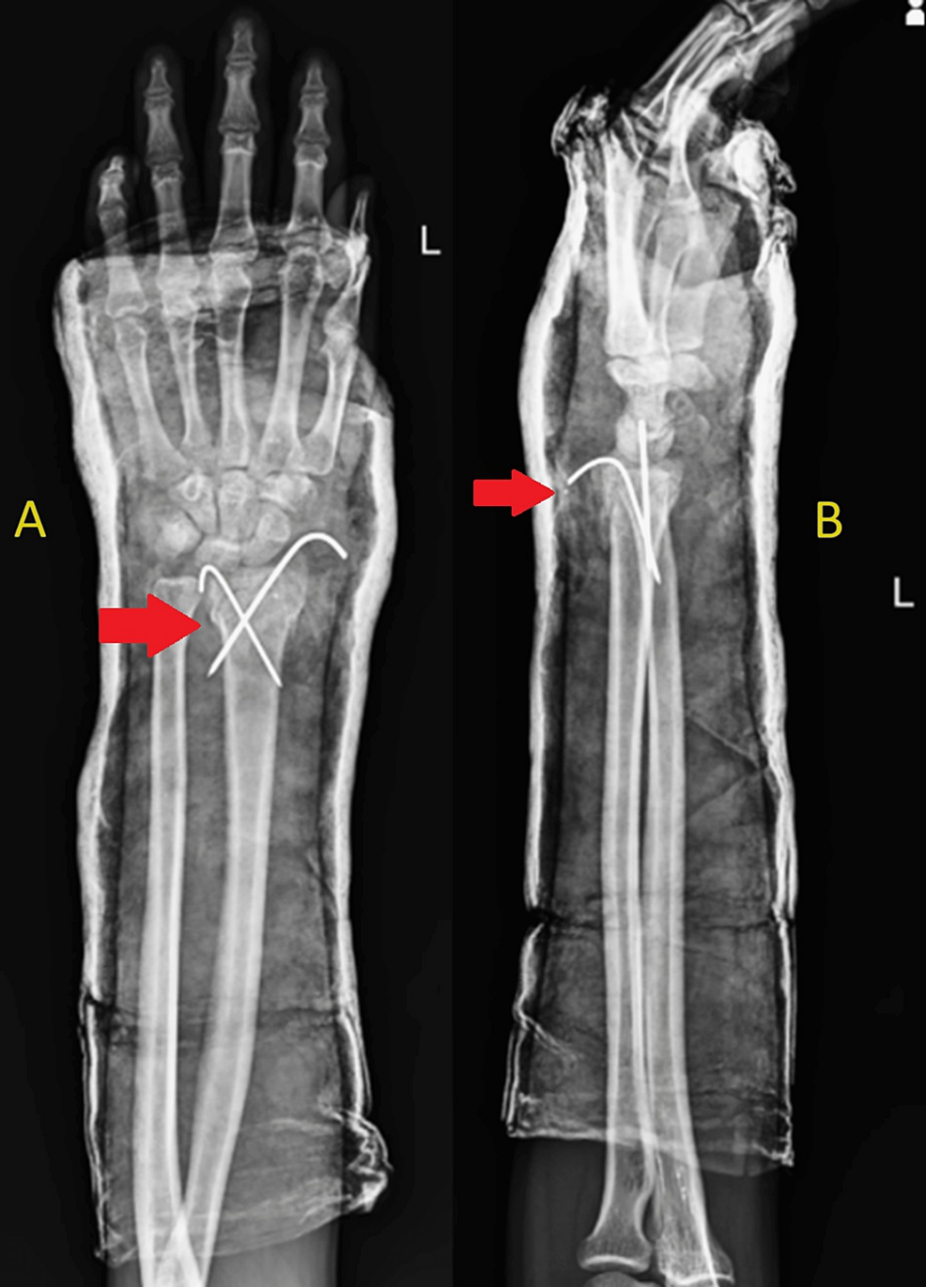
Immediate postoperative X-ray of the left wrist in anteroposterior (A) and lateral (B) views showing distal end of radius fracture with two K-wires in situ with overlying cast application. No signs of synovitis and juxta-articular osteoporosis.

His history was notable for surgery to treat a left-sided, distal-end radius fracture that took place in February 2021, which was managed with closed reduction and K-wire fixation (Figure 1). The patient was advised to remove the K-wire after six weeks, but the patient neglected the advice, and the K-wire was retained inside for six months. There was no past history of diabetes mellitus, tuberculosis, hypertension, or bronchial asthma. There was no history of substance abuse or stays in refugee camps, prisons, or night shelters. 

During the examination of the left hand, multiple wounds were seen on the dorsal aspect of the forearm near the wrist. A wound of 3x3 cm was present over the ulnar aspect of the wrist, characterized by oozing white and odorless discharge. There was also a 4x4 cm wound present over the radial aspect of the wrist, with caseous discharge oozing from the wound. Another wound on the wrist's dorsal aspect was 7x3cm in size, characterized by oozing white and odorless discharge.

**Figure 2:**
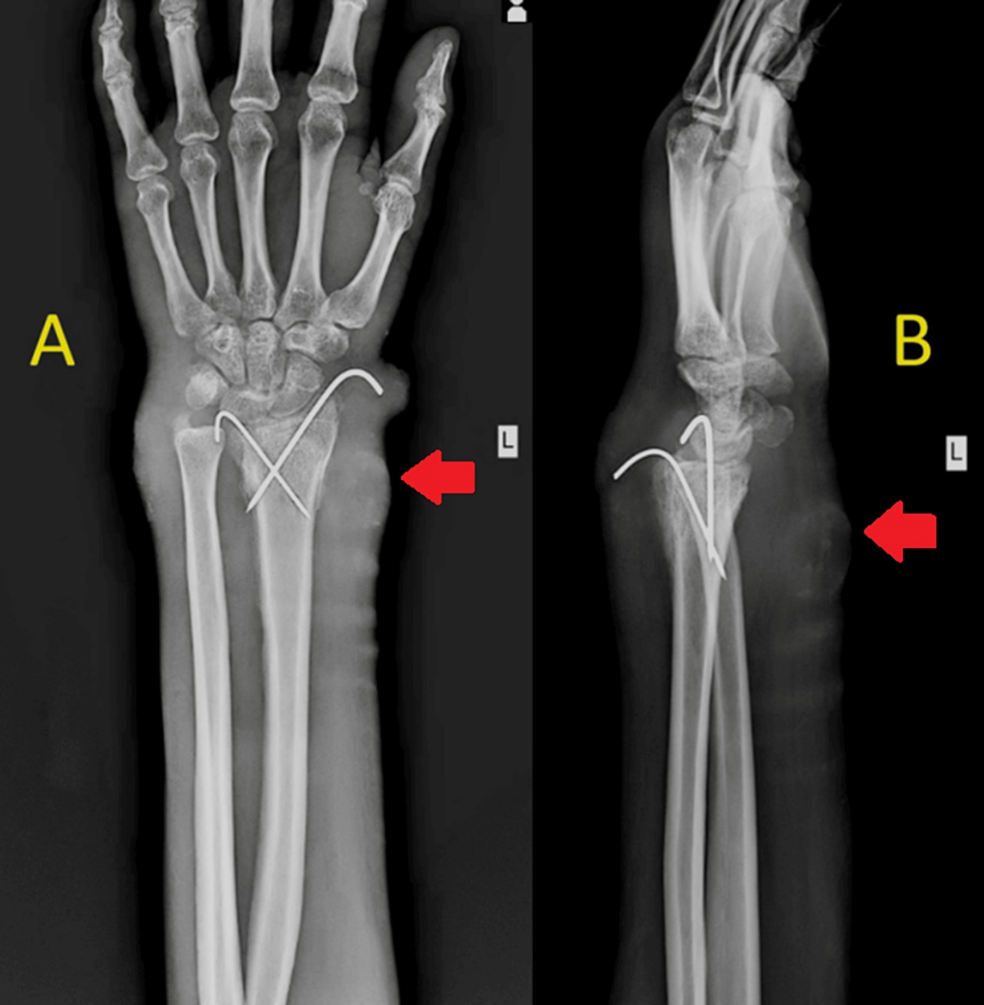
X-rays of left wrist anteroposterior (A) and lateral (B) views after six months showing the enlarged soft tissue shadow and sinus formation over the volar and dorsal aspect with callous seen along the medial, lateral anterior, and posterior cortex (red arrows) with two K-wires in situ.

The discharge was white in color and odorless. There was diffuse swelling present over the distal and volar aspect of the left wrist. A local rise in temperature was observed over the affected part. The fluctuation test yielded a positive result. No bony tenderness was elicited over the left wrist. Active wrist movements were painful and restricted. Active finger movements were present. Distal circulation was intact. Capillary refill was present. In the hematological investigations, the total white cell count was measured at 38,000/microliter, the erythrocyte sedimentation rate was found to be 45mm/h, and the C-reactive protein value was elevated at 455mg/L. In the blood culture and sensitivity test, the presence of *Staphylococcus aureus* was detected. On radiological investigations, a left wrist X-ray of the distal end of the radius fracture with callous showed the medial, lateral anterior, and posterior cortex with two K-wires in situ (Figure 2). The results of the chest X-ray were normal.

**Figure 3:**
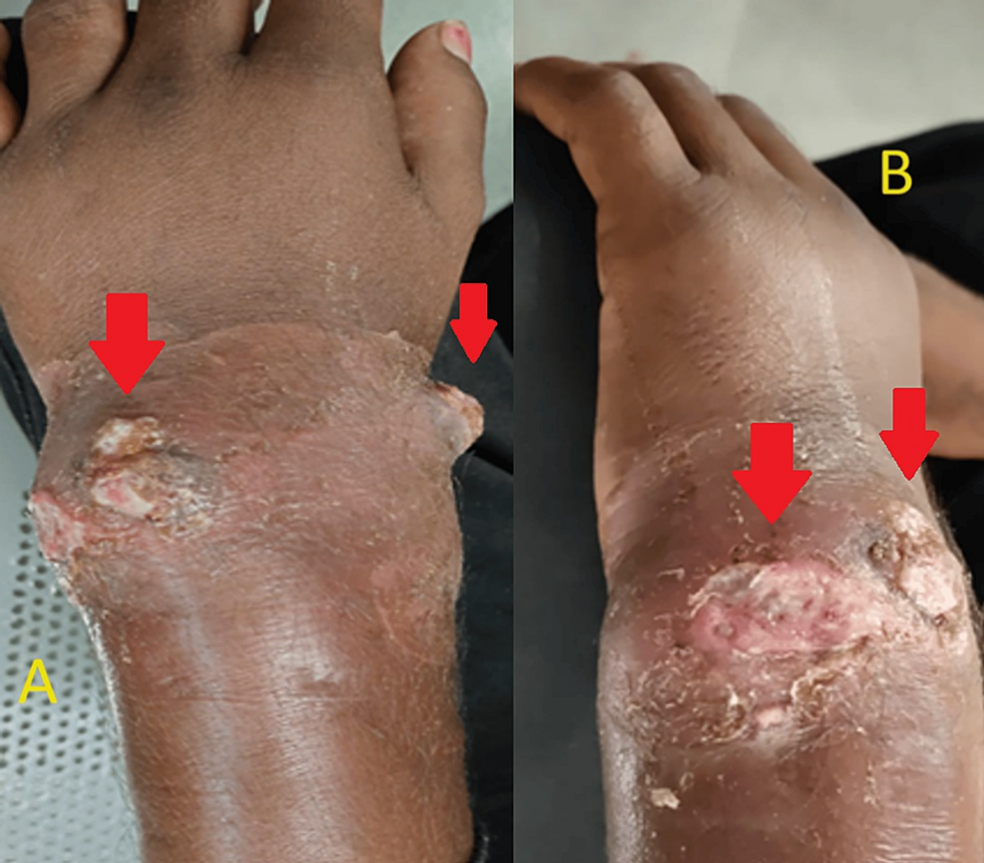
- A thick solid compressed caseous material discharge and sinus tract on left wrist joint seen on clinical examination A and B

He was diagnosed with discharging pin-site infection caused by the previous surgery performed six months ago i.e. distal end fracture of the left radius managed with two K-wire in situ. He was managed with K-wire removal followed by incision, drainage of abscess/ caseous material, and debridement of the site (Figure 3). A thick solid compressed caseous material was observed during the intra-operation. Intra-operative specimen samples were sent for microbiological examination and cartridge-based nucleic acid amplification test (CBNAAT). The pus culture and sensitivity tests were positive for methicillin-sensitive *Staphylococcus aureus*, and the Ziehl-Neelsen stain was positive for *Mycobacterium tuberculosis*. The CBNAAT report also returned positive for rifampicin-sensitive *Mycobacterium tuberculosis*. As extrapulmonary samples are paucibacillary, the ideal choice for testing would have been Xpert Ultra, a newer GeneXpert MTB/RIF Ultra assay (hereafter referred to as Xpert Ultra). This assay was been developed to overcome the limitations of the old Xpert MTB/RIF G4 assay, demonstrating improved sensitivity in the detection of TB and rifampicin resistance. However, due to our hospital's rural location, this test was unavailable, so we performed CBNAAT (Xpert MTB/RIF G4 assay).

**Figure 4:**
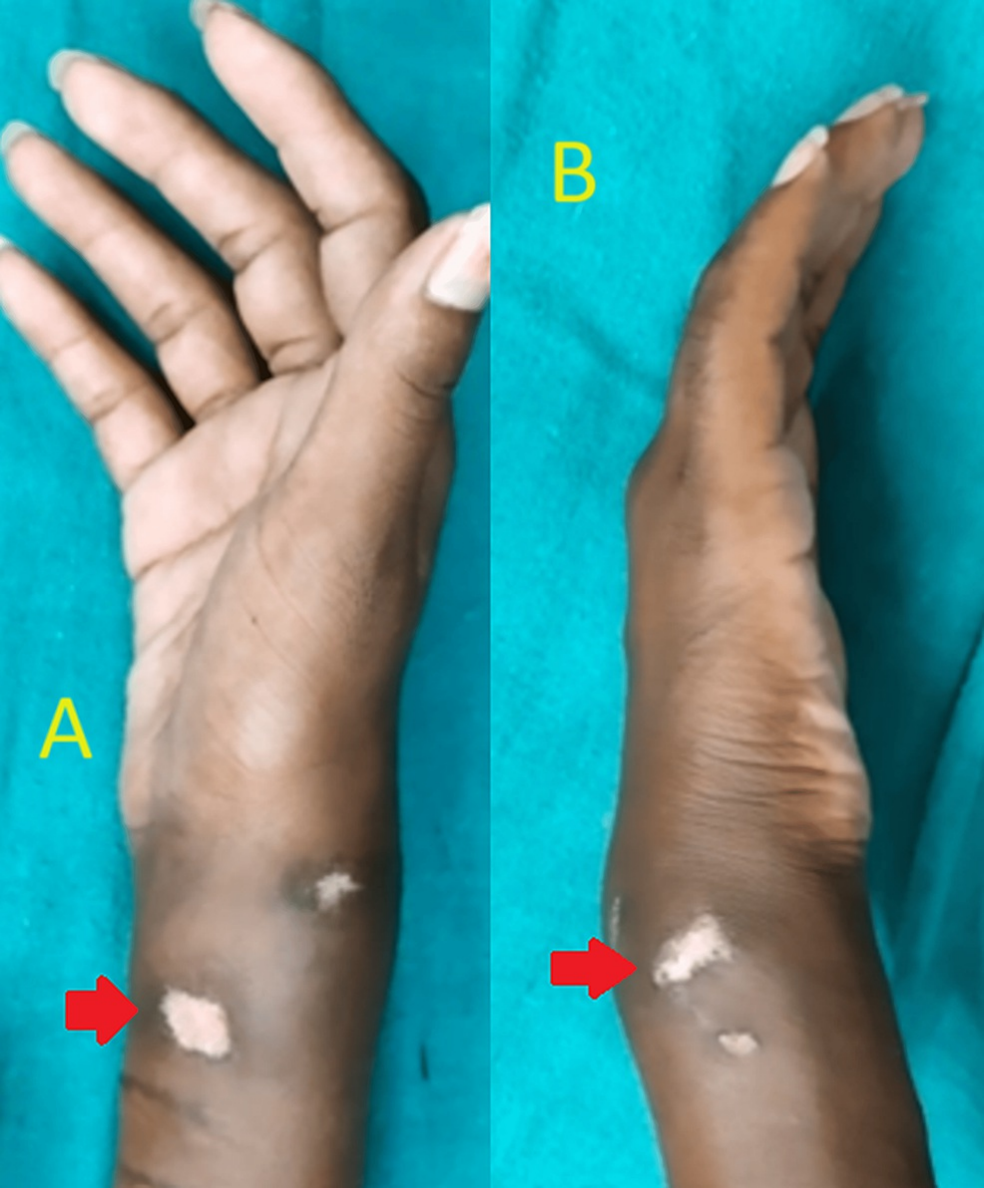
After 3 months of AKT treatment, the wounds were dried with no discharge A and B.

Based on the culture and sensitivity report, the patient was started on injected ceftriaxone + sulbactam (1.5gm) intravenously administered every 12 hours for five days. Based on the CBNAAT report, he was started on an anti-tuberculosis treatment (ATT; isoniazid 300mg/day + rifampicin 600mg/day + pyrazinamide 1500mg/day + ethambutol 1200mg/day) for six months and was discharged. The patient attended a follow-up visit after three months of ATT. After initiation of the ATT regimen, the inflammation and pain were reduced and the wounds were dry with no discharge (Figure 4). The hematological investigations found the total white cell count to be 11000/UL, the erythrocyte sedimentation rate was 12 mm/h, and the C-reactive protein value of 230mg/L was within normal limits (normal range of CRP: 0-3 mg/L).

**Figure 5:**
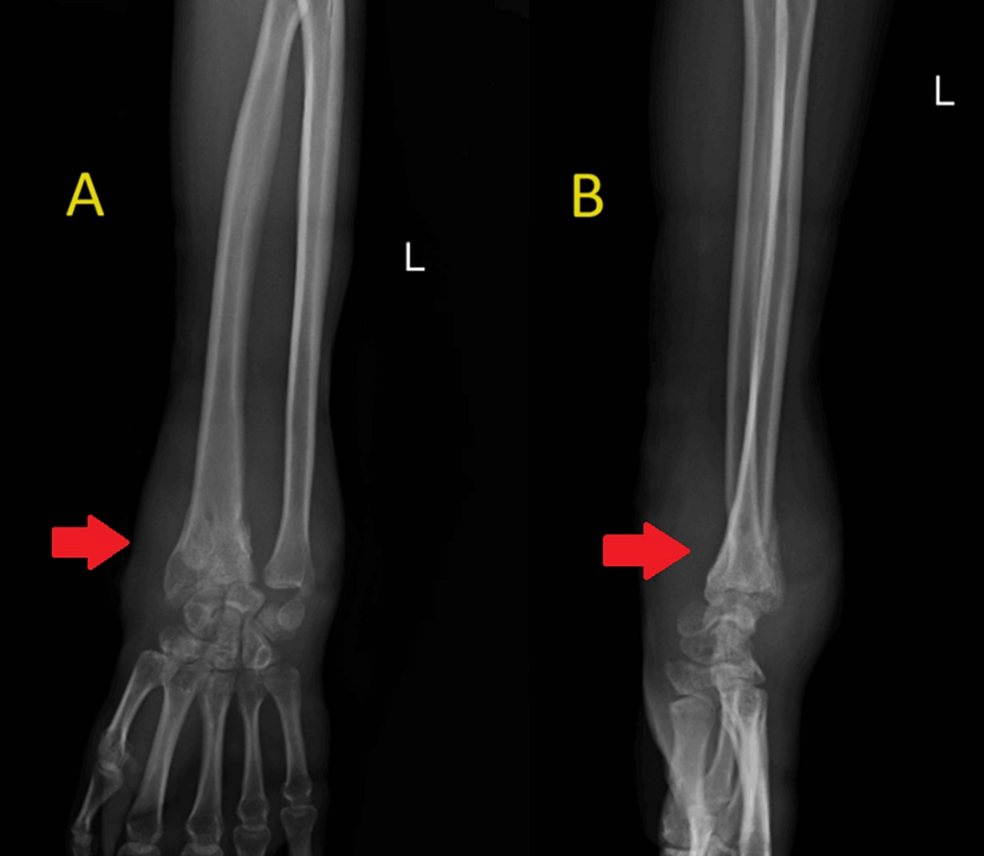
Post-operative X-ray of left wrist anteroposterior (A) and lateral (B) view after k wire removal, debridment, curettage, and sinus tract excision, showing no sign of infection

**Figure 6:**
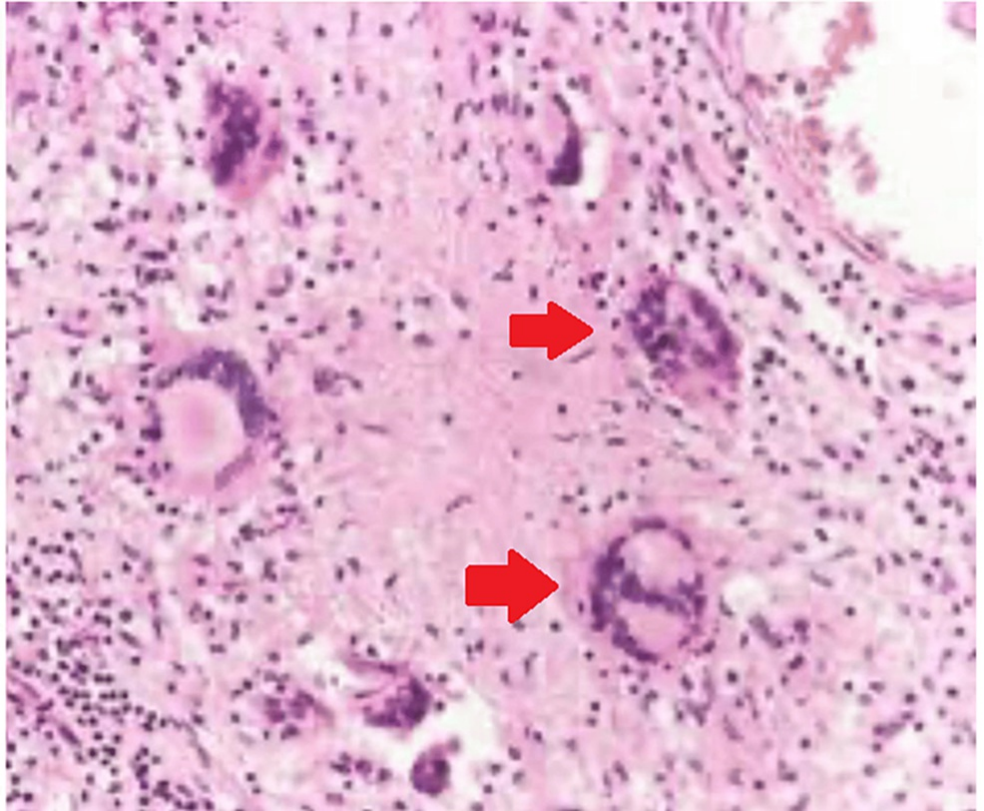
10x magnification stained with H and E showing caseous necrosis and granuloma.

**Figure 7:**
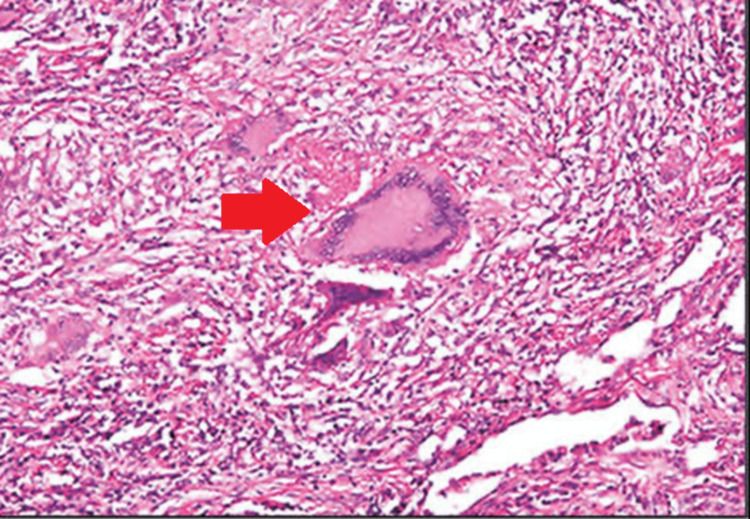
40x magnification of stained with H and E showing caseous necrosis, granuloma, and langhans giant cells.

A post-operative X-ray was done after the K-wire removal, debridement, curettage, and sinus tract excision (Figure 5). The histopathological report showed caseous necrosis and granuloma as seen in Figures 6-7.

## Discussion

In low-income countries, tuberculosis has long been a serious infection. The wide range of pathologies of osseous tuberculosis has several clinical and radiographical features and findings. Therefore, it is challenging to diagnose extrapulmonary TB [7]. Apart from this, there are various possible differential diagnoses such as subacute or chronic supportive arthritis, rheumatoid arthritis, benign bone tumor, osteochondrosis, and Kaposi sarcoma, making the path to a definite diagnosis challenging [8]. Additionally, the lesions may be mistaken as chronic osteomyelitis, which similarly exhibits bone damage. The treatment for cases with bone destruction involves eradication of infection by debridement of bone and if a defect is noted, with the use of an antibiotic cement spacer [9]. In the present paper, the case presented as a wrist abscess. If the concomitant infection is noted before the diagnosis is made, an antibiotic-impregnated medium has proven effective as it delivers a high concentration of antibiotics, which is sufficient for the treatment of local infection of the site [5].

Articular tuberculosis is a chronic condition with a poor prognosis which frequently impacts the joints that bear weight. The presentation of tuberculosis in small joints following an infected pin site is very rare, and because there is little reason to suspect the disease, the diagnosis is typically made later. Osteoarticular tuberculosis is the fourth most common type of extrapulmonary tuberculosis, behind localizations in the urogenital tract, ganglionic localization, and vertebral tuberculosis. The vertebrae alone are the most common location [7]. The gateways for extrapulmonary tuberculosis are primary foci reactivation and secondary dispersion through blood circulation [5].

Tenosynovitis is the major variety for the presentation of hand tuberculosis [4]. Typically, tubercular tenosynovitis develops gradually and progresses slowly. Generally, this condition presents with swelling along with mild pain and restriction in movements of the affected area. Without any other systemic indication of tuberculosis, the swelling moves through tendons. Common findings of hand tuberculosis are compound palmar or dumbbell ganglion of the ulnar bursa “sausage digit,” and carpal tunnel syndrome [4]. Tuberculosis of the wrist joint frequently develops swelling without tenderness, which tracks along tendons and is often associated with its systematic features [6]. However, in the above-presented case report, painful, increasing swelling without tendon involvement was the first symptom to appear, and it was accompanied by constitutional symptoms. As per the gold standard investigation for confirming tuberculosis, analysis of body fluid or tissue specimens is conducted to examine the histological pattern for the presence of acid-fast bacilli (AFB) [8]. For the diagnosis of tubercular arthritis, a classic triad of radiological findings (Phemister’s triad) can occur, including juxta-articular osteoporosis, peripheral bony erosion, and gradual joint space narrowing. However, this typical triad is not noted in every case. Therefore, there is a high chance that practitioners overlook the initial stages of tubercular arthritis due to the presence of non-specific clinical features [7]. When bone scans are done, there is an enhanced uptake seen, but this is not a particular clinical feature.

Tuberculin skin test (TST) or Mountox test is a supportive diagnostic test. Interferon-gamma release assays (IGRA) such as QuantiFERON TB Gold, are specifically employed for the detection of tuberculosis infection but are unable to discriminate between actively ongoing disease and latent infection of tuberculosis [6]. For the detection of amplified tuberculosis DNA, tests using polymerase chain reactions (PCR) are extremely sensitive but are unable to differentiate between live and dead bacilli. In culture-negative groups of cases, the PCR test results are 50-60% positive [9]. For the analysis of synovial fluid, bone, and soft tissue of joints, the PCR testing is more specific and faster [9]. Currently, synovial biopsies are the gold standard for diagnosing tubercular arthritis; they account for about 82% of cases and provide detailed characteristics of lymphocytes, large cells, and caseating granulomas in addition to caseation.

The treatment protocol for tubercular arthritis is easy compared to the classic management of osteoarticular tuberculosis in which only chemotherapy is required [9]. Nowadays, chemotherapy has been replaced by increasingly shorter treatment plans of six months due to the employment of more efficient medications for treatment [9]. Until the disappearance of clinical signs (up to three to four weeks), an orthopedic wrist splint should be maintained and followed by rehabilitation [5]. Anti-tubercular protocol drugs i.e., isoniazid, rifampicin, pyrazinamide, and ethambutol for six months are used for the treatment of localized tuberculosis. There are fewer requirements for surgery other than biopsy. To date, treatment options for this condition are still controversial. Surgical debridement is a debatable option for the treatment of wrist joint tuberculosis and the period of antitubercular chemotherapy is a less-noticed issue of discussion. Even if the antitubercular chemotherapy and debridement options are chosen, chemotherapy must be provided before any type of surgical debridement for the prevention of bony destruction and dissemination of disease [7]. A prolonged course of prescribed antitubercular chemotherapy should continue for nine or 12 months [9].

In our case report, atypical site of occurrence, clinical manifestation, and delay in biopsy led to a delayed diagnosis of wrist tuberculosis. If this case had been diagnosed earlier, anti-tubercular chemotherapy could have been initiated at the initial stage of manifestation, which would have reduced the need for repeated surgical debridement and yielded improved clinical outcomes.

## Conclusions

In conclusion, this case underscores the need for vigilance in orthopedic practice, revealing the challenges in recognizing atypical infections post-fracture with K-wire fixation. Collaboration among specialists is crucial for early detection and optimal management. The rarity of wrist tuberculosis following an infected pin site highlights the importance of ongoing research and awareness. This case prompts a reevaluation of postoperative monitoring strategies, emphasizing the significance of timely diagnosis and effective interdisciplinary treatment.
